# Inhibitory Receptor Trap: A Platform for Discovery of Inhibitory Receptors That Utilize Inositol Lipid and Phosphotyrosine Phosphatase Effectors

**DOI:** 10.3389/fimmu.2020.592329

**Published:** 2020-10-21

**Authors:** Bergren W. Crute, Rachel Sheraden, Vanessa L. Ott, Isaac T. W. Harley, Andrew Getahun, John C. Cambier

**Affiliations:** ^1^Department of Immunology and Microbiology, University of Colorado School of Medicine, Aurora, CO, United States; ^2^Department of Biomedical Sciences, National Jewish Health, Denver, CO, United States; ^3^Division of Rheumatology, Department of Medicine, University of Colorado School of Medicine, Aurora, CO, United States

**Keywords:** inhibitory receptor, SH2, ITIM, PD1, FcγRIIB, SHIP1, SHP1, SHP2

## Abstract

Among the areas of most impactful recent progress in immunology is the discovery of inhibitory receptors and the subsequent translation of this knowledge to the clinic. Although the original and canonical member of this family is FcγRIIB, more recent studies defined PD1 as an inhibitory receptor that constrains T cell immunity to tumors. These studies led to development of “checkpoint blockade” immunotherapies (CBT) for cancers in which PD1 interactions with its ligand are blocked. Unfortunately, although very effective in some patients, only a small proportion respond to this therapy. This suggests that additional as yet undescribed inhibitory receptors exist, which could be exploited. Here, we describe a new platform, termed inhibitory receptor trap (IRT), for discovery of members of this family. The approach takes advantage of the fact that many of the known inhibitory receptors mediate signaling by phospho-immunoreceptor tyrosine-based inhibition motif (ITIM) mediated recruitment of Src Homology 2 (SH2) domain-containing phosphatases including the SH2 domain-containing inositol phosphatase SHIP1 encoded by the INPP5D gene and the SH2 domain-containing phosphotyrosine phosphatases SHP1 and SHP2 encoded by the PTPN6 and PTPN11 genes respectively. Here, we describe the IRT discovery platform in which the SH2 domains of inhibitory phosphatases are used for affinity-based isolation and subsequent identification of candidate effectors *via* immunoblotting and high sensitivity liquid chromatography–mass spectrometry. These receptors may represent alternative targets that can be exploited for improved CBT. Salient observations from these studies include the following: SH2 domains derived from the respective phosphatases bind distinct sets of candidates from different cell types. Thus, cells of different identity and different activation states express partially distinct repertoires of up and downstream phosphatase effectors. Phosphorylated PD1 binds not only SHP2 but also SHIP1, thus the latter may be important in its inhibitory function. B cell antigen receptor signaling leads predominantly to CD79 mono-phosphorylation as indicated by much greater binding to LynSH2 than Syk(SH2)_2_. This balance of ITAM mono- versus bi-phosphorylation likely tunes signaling by varying activation of inhibitory (Lyn) and stimulatory (Syk) pathways.

## Introduction

Inhibitory receptors serve as key regulators of the immune system, terminating the immune response as appropriate, thus preventing the development of autoimmunity. Importantly, these controllers can be exploited therapeutically to enhance immunity to tumors and chronic infection (1, 2). While this so-called checkpoint blockade therapy (CBT) is very effective in some patients, only 15–20% benefit from the approach (3). While multiple factors play into the poor response rate, it may indicate that responses are limited by additional as yet undiscovered inhibitory receptors that have not yielded to discovery approaches employed to date. Elucidation of these may lead to more effective therapies. The work described herein seeks to define novel inhibitory receptors using an approach that identifies candidates based on their ability to engage inhibitory phosphatases that serve as the proximal downstream effectors of this receptor class.

The earliest described member of the immunoreceptor tyrosine-based Inhibition motif (ITIM)-containing inhibitory family is the low affinity IgG receptor FcγRIIB, which was initially shown by Phillips and Parker in 1984 to inhibit B cell antigen receptor signaling leading to blastogenesis (4, 5). Subsequent studies ascribed the point of B cell receptor (BCR) signal disruption as at or prior to phosphoinositide hydrolysis and calcium mobilization (6, 7). This inhibition occurred only when the two were co-aggregated. Later studies revealed that a 13 amino acid sequence in the cytoplasmic tail of the receptor contains all structural information required for engagement of inhibitory signaling machinery (8). With subsequent appreciation of other members of the inhibitory receptor family, a conserved sequence motif now known as the ITIM was recognized that occurs in most members of the family including in the 13aa FcγRIIB tail sequence (9, 10). The ITIM consensus motif consists of a tyrosine residue preceded by a -2 position hydrophobic amino acid and succeeded by a +3 position hydrophobic amino acid, therefore is I/VxYxxL. During signaling the conserved tyrosine is phosphorylated by Src family kinases. FcγRIIB ITIM tyrosine phosphorylation is mediated by Lyn that has been activated by BCR co-aggregation (11, 12). This relationship may explain the general requirement that to be activated most receptors in this family, e.g., PD1 and TIGIT, must be co-aggregated with a Src family kinase activating receptor such as BCR, T cell receptor (TCR), or FcγR1/2a/III/IV (13).

Inhibitory ITIM receptors described to date mediate signaling by recruitment of cytosolic inositol lipid and/or protein tyrosine phosphatases (10, 14, 15). This recruitment is mediated by phosphatase Src Homology 2 (SH2) domain binding to tyrosine phosphorylated ITIMs. The primary effector of FcγRIIB is the SH2-containing inositol lipid 5-phosphatase SHIP1 that contains a single SH2 that binds the pITIM (14, 16). SHIP1 would be expected to mediate inhibition of phosphoinositide breakdown by hydrolyzing PtdIns(3,4,5)P3 required for PLCγ translocation to the plasma membrane (17). Higher order BCR-FcγRIIB co-aggregation of these receptors can lead to recruitment of (SH2)_2_ -containing phosphotyrosine phosphatase SHP1 (18, 19). Indeed, most members of the inhibitory ITIM-containing receptor family signal by engaging SHP1 *via* dual pITIM binding to tandem SH2 domains (20). The best known exception is PD1, which preferentially binds SHP2 *via* the receptor’s pITIM and pITSM “switch” motif (15, 21). It appears that under some circumstances phosphorylated ITAMs (pITAMs) that normally transduce activating signals can recruit SHP1, at which point they have inhibitory function referred to as ITAMi (20, 22).

Activation of the tyrosine phosphatases is triggered by derepression resulting from tandem SH2 binding to dual pITIMs (21, 23–25). Interestingly, SHIP1 activation is triggered by phosphatase phosphorylation and interaction with partners such as DOK proteins (26). The consequence of these unique modes of activation is that SHP1 can only act locally on substrates within reach, while activated SHIP1 can inhibit responses to remotely stimulated receptors whose signaling requires generation and function of phosphatidylinositol 3,4,5 trisphosphate, the substrate of SHIP1 (27, 28). This occurs because SHIP1 can broadly reduce PtdIns3,4,5 levels globally, inhibiting signaling in trans.

Recruitment of SH2 containing phosphatases to membrane receptors is a shared inhibitory signaling mechanism among most immune system inhibitory receptors. While generally mediated by phosphorylated ITIMs, it appears that under appropriate conditions ITAMs and ITSMs can be inhibitory by use of this modality. It is known that both non-conserved sequences of the ITIM or ITAM and SH2 domains confer specificity in their interaction (29). In the example of SH2:ITAM interactions, the amino acids flanking the conserved tyrosines in the ITAM specify interactions with substrate, including phosphatases (29, 30). This specificity presumably underlies the non-redundant function of different ITIMs and ITAMs. We hypothesized that analysis of phosphatase SH2 domain binding specificity might be useful in resolving these relationships and identification of novel inhibitory receptors.

In this publication, we describe a method termed inhibitory receptor trap (IRT) for the isolation and identification of tyrosine phosphorylated surface receptors based on their binding to SH2 domains of specific downstream effector kinases and phosphatases. We have confirmed specific known inhibitory receptor—phosphatase interactions such as FcγRIIB : SHIP1SH2 and PD1:SHP2(SH2)_2_ using IRT. We also have identified CD79a association with the SH2 domains of inhibitory phosphatases which has been previous described. ITAMis are ITAMs that usually have embedded in them a sequence that shares great similarity to an ITIM allowing the recruitment of both activating and inhibitory signaling molecules (31). The significance of ITAMi containing receptors has been left largely uncharacterized and this approach may allow for identification and understanding of these molecules. We also demonstrate IRT capture of phosphorylated receptor proteins from cells stimulated *via* antigen receptor crosslinking. Utilizing cell surface biotinylation, IRT, and liquid chromatography–mass spectrometry (LC-MS/MS) in combination allows high throughput and sensitive probing of tyrosine phosphorylated receptor interactions with their cognate SH2-containing effectors.

## Material and Methods

### Mice

In all cases, 8–10-week-old female C57BL6/J mice purchased from Jackson Laboratories were used to obtain indicated immune cell populations. Mice were housed in the Animal Research Facilities at the University of Colorado Anschutz Medical Campus. All experiments were performed in accordance with the regulations and approval of University of Colorado Institutional Animal Care and Use Committee.

### Cell Purification

Spleens were dissociated in complete medium [IMDM (HyClone), 5% FBS (Sigma), 1 mM sodium pyruvate (HyClone), 2 mM L-glutamine (Corning), 50 μM β-mercaptoethanol (Sigma), 50 μg/mL of Gentamicin (Gibco), and 100 U/mL of penicillin/streptomycin (Gibco)], and red blood cells removed using ammonium-chloride-potassium (ACK) lysis. Naive splenic B cells were isolated using negative selection with anti-CD43 (Ly-48) MicroBeads (Miltenyi Biotec 130-049-801). CD3^+^ splenic T cells were purified by negative selection using the Pan T cell Isolation Kit II (Miltenyi Biotec 130-095-130).

### Cell Culture and Activation

A20 cells were cultured in RPMI (Corning) containing 10% FBS, 1mM sodium pyruvate, 2mM L-glutamine, 50 μM β-mercaptoethanol, 50 μg/mL of Gentamycin, and 100 U/mL of penicillin/streptomycin. RBL-2H3 cells were cultured in MEM (Corning) containing 10% FBS, 2mM L-glutamine, and 100 U/mL of penicillin/streptomycin. RAW264.7 cells were cultured in DMEM high glucose (Corning) containing 10% FBS 1 mM sodium pyruvate, 2 mM L-glutamine, and 100 U/mL of penicillin/streptomycin. CD3+ T cells were either used directly *ex vivo* (naïve) or cultured at 1E6/mL in complete medium on plates pre-coated with 5 μg/mL of anti-CD3 (clone 145-2C11, BioLegend) and supplemented with soluble 0.5 μg/mL of anti-CD28 (clone 37.51, BioLegend) and 12.5 U/mL of IL-2 (Roche 11271164001) for 72 h to induce PD1 expression. PD1 expression was confirmed *via* flow cytometry staining (**Supplementary Figure 1**) with anti-PD1 PE (clone J43, eBioscience) anti-CD4 FITC (clone GK1.5, BD Biosciences), and anti-CD8 APC (clone 53-6.7, BD Biosciences).

### Acute Cell Stimulation

In all cases, cells were acutely stimulated in serum free complete medium. 10E6 Purified B and T cells at 20E6 cells/ml were stimulated with 100 μM pervanadate (PV) for 5 min at 37°C. Anti-mouse IgG H+L (Zymed 61-6500) or F(ab’)_2_ anti-mouse IgG H+L (Jackson ImmunoResearch 315-006-003) were used at a final concentration of 10 μg/mL or 6.4 μg/mL to stimulate B cells for 5 min at 37°C. When indicated cells were surface biotinylated using EZ-Link™ Sulfo-NHS-LC-Biotin (Thermo Scientific A39257) as per manufacturers protocol. After stimulation cells were pelleted by centrifugation at 6000rpm for 30 seconds in a tabletop centrifuge. Supernatant was aspirated and cells were lysed by resuspension in cold lysis buffer composed of 25 mM Tris-HCl (pH 7.4), 150 mM NaCl, 1% NP-40, 1 mM EDTA, 1 mM DTT, and 5% glycerol supplemented with protease and phosphatase inhibitors (100 µg/mL aprotinin, 100 µg/mL α-1-antitrypsin, 100 µg/mL leupeptin, 1 mM PMSF, 10 mM NaF, 2 mM NaVO_3_, and 10 mM tetrasodium pyrophosphate). Lysates were incubated on ice for at least 30min then cleared of particulate debris by centrifugation at ≥16,000g for 10min at 4°C. Cleared lysates were stored at -80°C.

### Recombinant SH2 Domain Production and Purification

In brief, cDNAs encoding LynSH2, SHIP1SH2, SHP1(SH2)_2_, and SHP2(SH2)_2_ domain(s) were prepared from mouse B cells using PCR previously described (25, 32). Mouse cDNA sequences for Syk ENSMUST00000120135.7 were obtained from Ensembl. SH2 domains were defined according to UniProt entry P48025. SYK(SH2)_2_ (1–831 bp) gBlock was purchased from IDT with engineered sites for restriction cloning. SHIP1SH2, SHP1(SH2)_2_, and SHP2(SH2)_2_ were cloned into the pGEX-5x-1 vector (GE Healthcare) for expressing recombinant GST fusion proteins. LynSH2 and SYK(SH2)_2_ were cloned into the pGEX-6P-1 vector (GE Healthcare) for expressing recombinant GST fusion proteins. DH5α *Escherichia coli* were transformed with pGEX vectors containing SH2 domains for plasmid production. Rosetta II *E. coli* were transformed with SH2 domain-containing pGEX vectors for robust production of recombinant proteins. Cultures were inoculated with a single colony of Rosetta II *E. coli* containing SH2 domain pGEX vectors and grown overnight (16 h) at 37°C. 10mL of overnight culture was used to seed 1L of LB broth and allowed to grow for 3–4 h at 37°C reaching an OD600 ~0.6. Protein production was induced by addition of 0.25 mM IPTG for 3 h at 37°C. Bacteria were harvested by centrifugation at 4,000g for 10 min and lysed using previously established protocols for SH2-GST fusion protein production (25). Fusion proteins were isolated by GSH-Sepharose chromatography (GE Healthcare) and subsequently cleaved either on column or in solution using Factor Xa (NEB) or HRV3C (Pierce) proteases. Cleaved protein preparations were depleted of GST by 3x adsorption using a GSH column. Purity was assessed using SDS-PAGE with Coomassie staining.

### Recombinant SH2 Domain Conjugation to Sepharose Beads

In brief, CNBr-activated Sepharose 4B beads (GE Healthcare) were washed with 1 mM HCl for 10 min to achieve activation for coupling. Purified SH2 domains that had been dialyzed into 0.1 M NaHCO_3_ (pH 8.3) containing 0.5 M NaCl were added to the activated Sepharose beads. Beads were conjugated with excess protein (1 mg protein/0.04g of CNBr Sepharose) overnight at 4°C while rotating. Beads were then incubated with 100 mM Tris HCl (pH 8.0) for 2 h at room temperature to quench unreacted groups. Conjugated beads were washed with 3 cycles of alternating pH buffers [each cycle consists of 100 mM sodium acetate (pH 4.0) containing 0.5 M NaCl and then 0.1 mM Tris-HCl (pH 8.5) containing 0.5 M NaCl]. Finally, conjugated beads were washed and stored in PBS + 0.02% sodium azide at 4°C.

### Immunoprecipitation and SH2 Domain Enrichment (IRT)

Immunoprecipitation was performed with anti-PD1 clone J43 (eBioscience) and anti-FcγRIIB, clone 2.4G2. In brief, 0.5 μg of antibody/1E6 cells/100 μL was added and incubated for 1 h while rotating. Protein G beads (Life Technologies) that had been washed 3x in lysis buffer were added to lysates (500 μL of lysates/30-μL beads) and incubated for 2 h. Protein G beads were then washed 3x in lysis buffer and adsorbed protein eluted by boiling in reducing Laemmli SDS-PAGE sample buffer for 10 min. SH2 domain-conjugated Sepharose beads were washed 3x in lysis buffer before being added to cell lysates (500 μL of lysate/35-μL beads) and rotated at 4°C for 2 h. Beads were then washed 3x with lysis buffer and eluted by boiling in reducing Laemmli SDS-PAGE sample buffer for 10 min. 2-step enrichment was performed using SH2 domains as described before and eluting them 3x with 100 μL of 100 mM glycine (pH 2.5) for 5 min each. Low pH glycine eluates were combined and immediately neutralized by addition to 30 μL of 1 M TrisHCl (pH 8.5). 30 μL of streptavidin agarose (Thermo Scientific 20347) was then washed with PBS and added to each sample and rotated for 2 h at 4°C. Streptavidin agarose was washed 3x with lysis buffer and eluted by addition of reducing Laemmli SDS-PAGE sample buffer and boiled for 10 min.

### Immunoblotting

Whole Cell lysates, immunoprecipitates or SH2 binding proteins were fractionated by SDS-PAGE and transferred to PVDF using semi-dry blotting conditions. PVDF was then blocked using either 3% BSA TBST [10 mM Tris-HCl (pH 8.0), 150 mM NaCl, and 0.05% Tween] or 5% non-fat dry milk TBST. Antibodies used to blot included: anti-pTyr clone 4G10, anti-PD1 (R&D Systems AF1021), anti-pCD79a Y182 (Cell Signaling Technology 5173S), anti-pFcγRIIB Y292 (Abcam EP926Y), and anti-β-actin (clone C4, Santa Cruz). In addition, we used rabbit polyclonal anti-FcγRIIB cytoplasmic tail, anti-CD22 cytoplasmic tail, and anti-CD79a raised in our laboratory as described previously (26, 32). Secondary antibodies used included anti-mouse IgG Trueblot HRP (clone eB144, Rockland), anti-rabbit IgG Trueblot HRP (clone eB182, eBioscience), anti-rabbit IgG HRP (Cell Signaling Technology 7074S), and anti-goat IgG HRP (R&D Systems HAF109). Blots were incubated in Pierce SuperSignal West Pico PLUS HRP (Thermo Scientific) substrate and visualized on a G:BOX by Syngene. Subsequent immunoblot images were quantified using Image Studio Lite ver 5.2 (LI-COR).

## Results

### Design of the Inhibitory Receptor Trap (IRT) Platform

The goal of these experiments was to develop a novel approach for isolation of inhibitory receptors based on their ability to bind the SH2 domains of the inhibitory phosphatases. This method could be used for identification of new therapeutic targets for CBT and for characterization of the receptome of immune cell populations.

**Figure 1:**
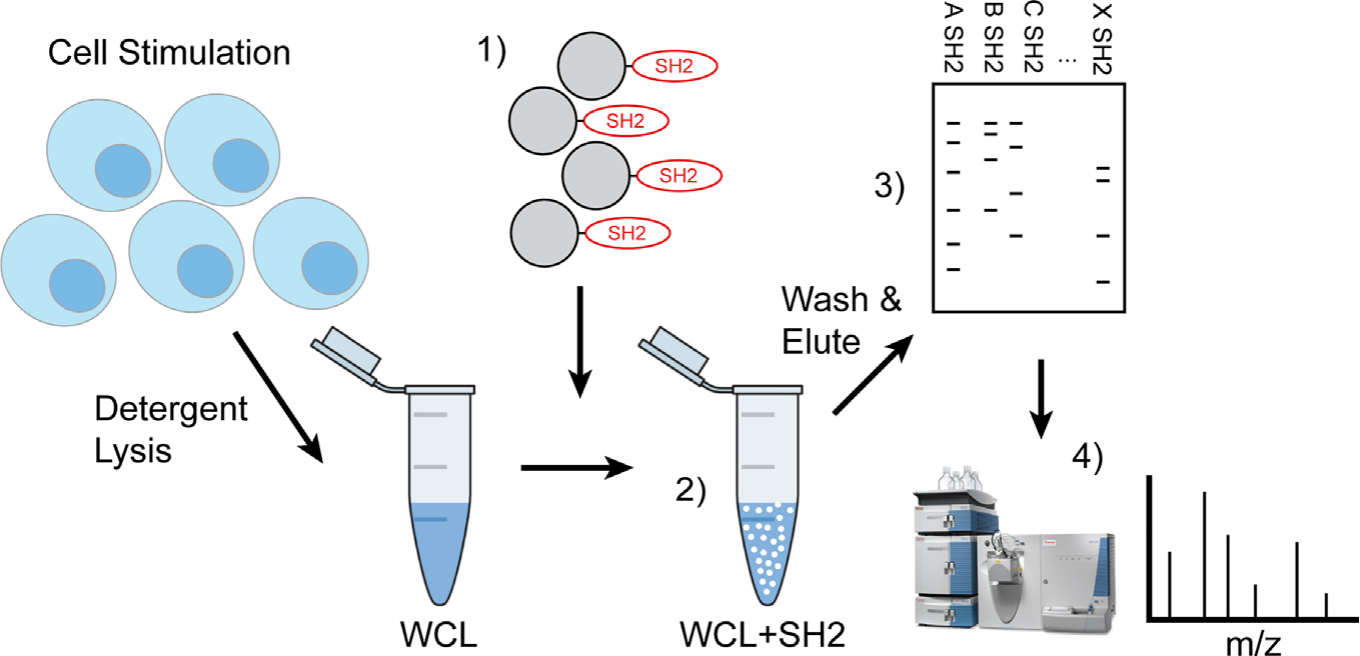
Workflow of IRT (Inhibitory Receptor Trap). Cells were stimulated to induce phosphorylation of inhibitory receptors *via* chemical inhibition or antigen receptor cross linking and detergent lysates made. 1) Pre-washed SH2 domain conjugated beads were added to cleared cellular lysates 2) to capture phosphorylated proteins then washed to remove nonspecific interacting proteins and eluted using reducing Laemmli buffer. 3) Eluted phosphoproteins were subjected to SDS-PAGE electrophoresis and then transferred to PVDF for western blotting to define candidates or 4) stained bands were excised for analysis by LC-MS/MS.

These phosphatases, the SH2-containing inositol 5-phospatase SHIP1, the SH2-containing protein phosphotyrosine phosphatase SHP-1, and SH2-containing protein phosphotyrosine phosphatase SHP-2, have been shown previously to function as the proximal effectors of ITIM-containing receptors. As shown in **Figure 1**, the approach taken for development of IRT involves initial stimulation of cells under conditions that induce tyrosine phosphorylation of ITIMs. These can include conditions that induce phosphorylation of substrates nonspecifically by inhibiting the phosphotyrosine phosphatases that normally balance their phosphorylation or conditions in which phosphorylation is stimulated by specific ligands. For the former, we utilized pervanadate stimulation. For the latter, we utilized antibodies against antigen receptors. Stimulation was followed by detergent lysis using NP40. As indicated, SH2 domain-conjugated Sepharose beads were added to cleared whole cell lysates to capture tyrosine phosphorylated binding partners. After washing, the beads were treated with reducing Laemmli buffer to elute SH2 bound proteins. Eluates were fractionated by SDS PAGE and 1) transferred electrophoretically to PVDF membrane that was then immunoblotted to define candidates, or 2) Comassie stained bands were excised from gels for analysis by LC-MS/MS. The latter approach provides for the unsupervised identification of interacting proteins. In this publication we have forgone presentation of LC-MS/MS analysis and focus on proving the effectiveness of our approach to identify known SH2 interactions.

### Distinct SH2 Domain Adsorbents Capture Distinct Phosphorylated Proteins

Cells of the immune system utilize distinct inhibitory receptor–phosphatase effector pairs to control cellular activation and function. The specificity of these interactions of receptor effector pairs is determined by SH2 domain unique recognition of phosphotyrosine in the context of ITIM sequence (x) flanking conserved residues in the I/VxYxxL ITIM. We first wanted to determine if SH2 domains derived from different effector phosphatases and kinases bind distinct phosphoproteins. To address specificity, we developed SH2 domain probes from the activating tyrosine kinases Lyn and Syk and inhibitory phosphatases SHIP1, SHP1 and SHP2 and subjected pervanadate stimulated lysates from different cell types to IRT enrichment. Since the tandem SH2 domains found in Syk, SHP1, and SHP2 may bind cooperatively, the probes derived from these proteins contained both SH2 domains. We employed the single SH2 domains found in Lyn and SHIP1.

**Figure 2:**
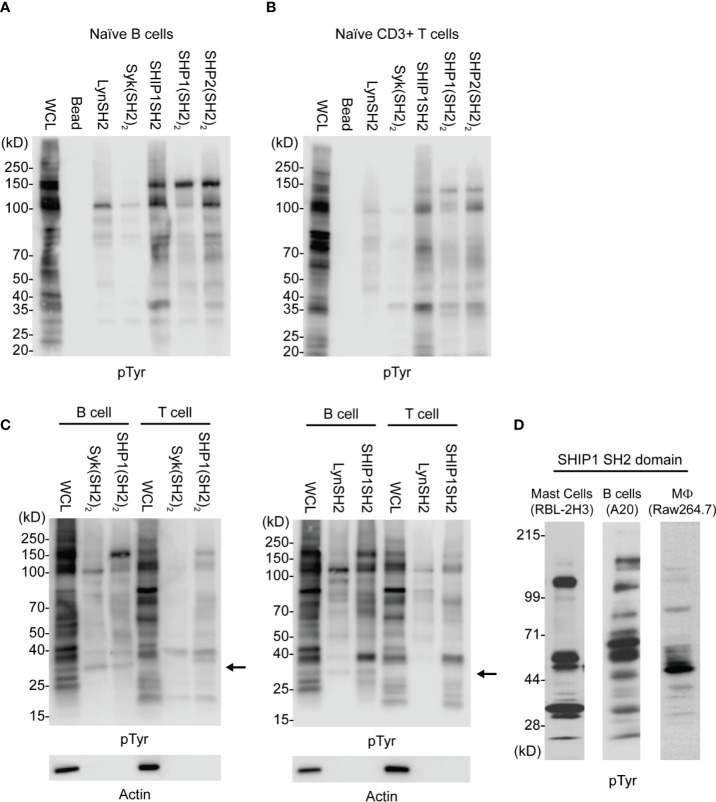
Comparison of kinase and phosphatase SH2 binding proteins from PV stimulated cells. Cellular lysates derived from **(A)** splenic B cells and **(B)** splenic T cells enriched for phosphorylated tyrosine containing molecules using SH2 domain reagents derived from activating and inhibitory molecules. **(C)** Direct comparison between B and T cells using either tandem (SH2)_2_ or single SH2 reagents. **(D)** Cellular lysates from indicated cell lines show differential enrichment for phosphoproteins when subjected to IRT using SHIP1SH2. Immunoblots are representative of *n* = 3 independent experiments.

As seen in **Figures 2A–C**, SH2 domain adsorbents bind only a small proportion of tyrosine phosphorylated proteins detected in whole cell lysates, indicating some level of selectivity. Further, kinase SH2 adsorbents and phosphatase SH2 adsorbents bound largely distinct sets of tyrosine phosphoproteins in lysates of naïve B cells and naïve T cells. Interestingly, findings suggest that Lyn and Syk may have some interactions in common, and the three phosphatases may have some common interactions distinct from the kinases. These results demonstrate that different SH2 domains, whether singular or tandem, have unique specificity. Furthermore, as shown in **Figure 2C** wherein binding of B cell and T cell phosphoproteins was compared directly, these cells have partially distinct repertoires of phosphoproteins that bind to each of Lyn, SHIP1, Syk and SHP1. Finally, shown in **Figure 2D** is a comparison of IRT enrichment of SHIP1SH2 binding phosphoproteins from three different cell lines representing basophils (RBL-2H3), B cells (A20), and macrophages (RAW264.7). Anti-phosphotyrosine blotting of enriched proteins revealed both distinct and shared interactions across cell types supporting the existence of cell type specific inhibitory interactomes.

**Figure 3:**
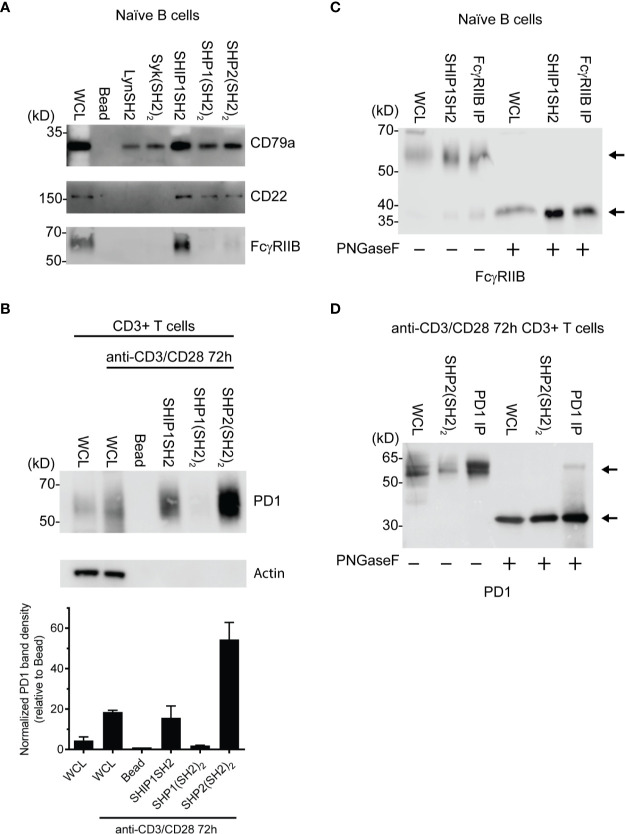
Identification of specific SH2 substrates in PV stimulated cells **(A)** CD79a, CD22, and FcγRIIB are known tyrosine phosphorylated receptors that recruit SH2 domains. **(B)** (Top) Lysates of splenic T cells activated for 72 h with anti-CD3/CD28 were probed with SH2 domains from indicated molecules. PD1 is enriched specifically by the SHP2(SH2)_2_ and SHIPSH2. (Bottom) Quantification of PD1 staining normalized to the bead control. Data is plotted from 3 independent experiments. **(C, D)** Cells were stimulated with PV prior to treatment with N-glycanase. FcγRIIB and PD1 have observed molecular weight shifts post N-glycanase treatment in WCL, SH2 reagent, and immunoprecipitation lanes. Bars in **(B)** represent ± SEM; immunoblots are representative of *n* = 3 independent experiments.

To further explore the utility of IRT, we tested its ability to detect previously demonstrated interactions between receptors and specific phosphatases and kinases. As expected, FcγRIIB was bound by SHIP1 (**Figure 3A**) consistent with the dominant role of SHIP1 in inhibitory signaling by this receptor (14, 16). The SHIP1SH2 IRT sample was subjected to mass spectrometric analysis, which confirmed the presence of FcγRIIB (data not shown). As expected, SHIP1, SHP1, and SHP2 SH2 domains all bound CD22 (33). Interestingly, all SH2 domains tested bound CD79a (**Figure 3A**). This was expected of Lyn, Syk, and SHIP1 (34, 35) and heavily suggested for SHP1 (36), but interactions between CD79a and SHP2 have not been demonstrated previously.

We next explored the ability of IRT to detect the previously demonstrated interaction between SHP2 and PD1 (15). In this experiment we utilized splenic T cells that were first activated by culture for 72 h with anti-CD3 and anti-CD28 antibodies and then stimulated acutely with pervanadate before lysis. Lysates were subjected to enrichment using SH2 domain adsorbents. As shown in **Figure 3B**, PD1 was bound by the (SH2)_2_ domain of SHP2. SHP2(SH2)_2_ and SHP1(SH2)_2_ IRT samples were subsequently subjected to mass spectrometric analysis, which confirmed PD1 enrichment by SHP2(SH2)_2_ and lack of enrichment by SHP1(SH2)_2_ (data not shown). Surprisingly, PD1 was also bound by the SH2 of SHIP1. This may indicate that SHIP1 functions as an alternative PD1 effector, the existence of which has been suggested by studies showing that PD1 mediated inhibition is partially preserved in the absence of SHP2 expression (37).

Further confirmation of the identity of PD1 and FcγRIIB among proteins that engage SH2 domain adsorbent was generated by analysis of the core protein mass generated by PNGaseF treatment of adsorbates prior to SDS-PAGE, transfer and blotting. PNGaseF treatment resulted in a molecular weight shift of the presumptive FcγRIIB from ~60 kDa to weight of 37 kDa, consistent with core protein mass (**Figure 3C**). PNGaseF treatment of SHP-2 adsorbates of activated T cells yielded a molecular weight shift of presumptive PD1 from 55–60 kDa to the core molecular weight of 32 kDa as would be expected (**Figure 3D**). Observation of molecular shifts for FcγRIIB and PD1 increases our confidence in prior observations made by immunoblotting. In addition, this experiment demonstrates that N-glycanase treatment is useful in generation of more defined bands and core protein mass, and is therefore helpful in protein identification.

### IRT Capture of Phosphatase Binding Partners Following Physiologic Phosphorylation

While pervanadate induced protein tyrosine phosphorylation is useful in defining SH2-phosphoprotein interactions that can occur in an unbridled situation in which phosphorylation is super-physiological, it is important to be able to detect interactions that occur upon specific receptor stimulation. Further, the latter allows analysis of requirement for co-aggregation to induce phosphorylation of sites required for phosphatase interactions.

**Figure 4:**
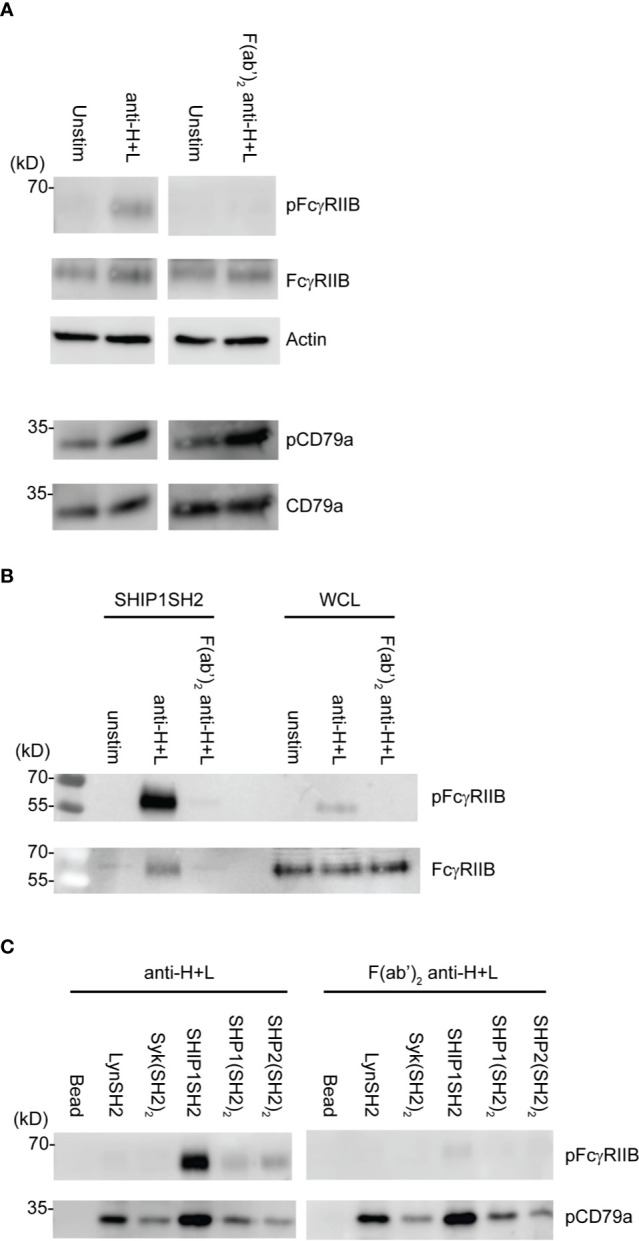
Physiologic stimuli induce phosphorylation of physiologic targets which bind specific SH2 domains. *Ex vivo* splenic B cells were stimulated with either 10 μg/mL of anti-IgG H+L or 6.4 μg/mL F(ab’)_2_ anti-IgG H+L per condition. **(A)** 5E5 cell equivalents of WCL from unstim and anti-IgG H+L or F(ab’)_2_ anti-H+L were loaded and run by SDS-PAGE and blotted for pFcγRIIB, FcγRIIB, pCD79a, CD79a, and Actin. **(B)** 10E6 cells were left unstimulated, stimulated with anti-IgG H+L, or F(ab’)_2_ anti H+L, lysates were subjected to pull down using SHIP1SH2 reagent. Eluted proteins were run with 2E6 cell equivalents of WCL per condition and blotted for pFcγRIIB and total FcγRIIB. **(C)** 10E6 cells were stimulated with anti-IgG H+L and F(ab’)_2_ anti-H+L and subjected to pull down using SH2 domain reagents. Adsorbed proteins were eluted, run on SDS-PAGE and blotted for pFcγRIIB and pCD79a. Immunoblots are representative of *n* = 3 independent experiments.

Induction of FcγRIIB ITIM phosphorylation requires its co-ligation with antigen or activating IgG Fc receptors. To test physiologic requirements for induction of FcγRIIB competence to bind effector phosphatases detectably, we stimulated naïve B cells with equimolar amounts of affinity purified rabbit anti-IgG Heavy and Light chain antibodies to co-aggregate FcγRIIB and the BCR, or F(ab’)_2_ fragments of the same antibody to stimulate only BCR. Whole cell lysates were generated and blotted with tyrosine phosphospecific antibodies against FcγRIIB and CD79a. As shown in **Figure 4A**, stimulation with either antibody induced phosphorylation of CD79a detectable in whole cell lysates. As expected, phosphorylation of FcγRIIB was stimulation by anti-H+L but not F(ab’)_2_ anti-H+L. Phosphorylation of FcγRIIB by anti-H+L stimulation led to abundant FcγRIIB enrichment *via* SHIP1SH2 reagent (**Figure 4B**). The specificity of the SHIP1SH2 reagent for phosphorylated FcγRIIB was indicated by absence of FcγRIIB enrichment in unstimulated and F(ab’)_2_ anti-H+L conditions. We then determined the ability of IRT to capture FcγRIIB and CD79a whose phosphorylation was stimulated by these ligands (**Figure 4C**). Receptor co-stimulation with IgG anti-H+L led to efficient capture of pFcγRIIB by SHIP1SH2 reagent. Interestingly, although to a lesser degree, pFcγRIIB was also captured by SHP1 and SHP2(SH2)_2_ adsorbents, consistent with the function of these phosphatases in inhibitory pFcγRIIB signaling (10). When FcγRIIB was not co-engaged with BCR, in the case of F(ab’)_2_ anti-H+L stimulation, only basal pFcγRIIB could be detected in SHIP1SH2 adsorbents. Reminiscent of findings in **Figure 3**, all adsorbents captured phosphorylated CD79a, albeit with differing efficiency. It is curious that the Syk(SH2)_2_ bound less pCD79a than the LynSH2. It is known that Syk binding to CD79 requires phosphorylation of both ITAM tyrosine residues, while Lyn binding does not (38, 39). This result may indicate that anti-BCR stimulates primarily monophosphorylation of CD79 ITAMs. We conclude from these experiments that SH2 domain adsorbents are able to capture receptors phosphorylated upon B cell antigen receptor crosslinking.

### Refinement of IRT to Identify Cell Surface Binding Partners

As utilized to this point, phosphoproteins identified by IRT could be localized on the cell surface or may be intracellular. To focus the IRT only on identification of cell surface binding partners, we utilized cell surface biotinylation as an additional filter.

**Figure 5:**
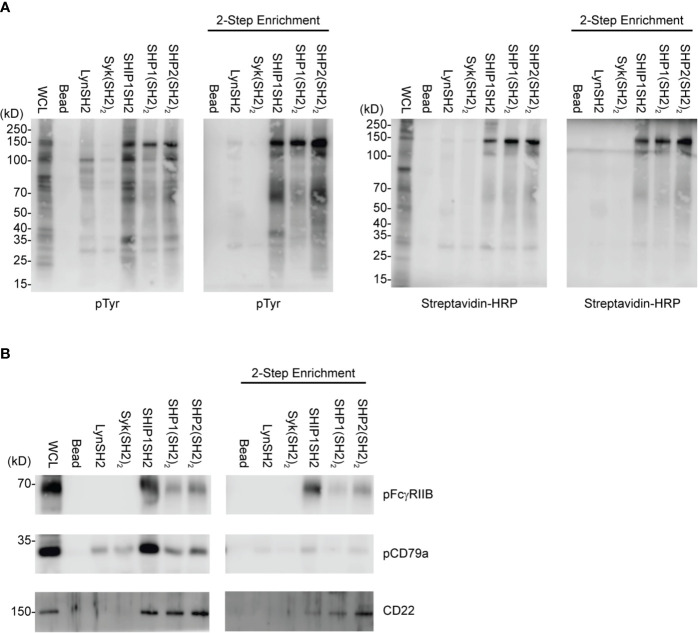
IRT Refinement with cell surface biotinylation. Splenic B cells were cell surface biotinylated, PV stimulated, and subjected to either an SH2 domain pulldown or SH2 domain pulldown plus avidin enrichment. **(A)** Using this two-step method we can purify specific SH2 binding receptors. **(B)** pFcγRIIB, pCD79a, and CD22 were examined as specific SH2 domain substrates for their presence in two-step enrichment. Immunoblots are representative of *n* = 3 independent experiments.

In this proof of concept experiment, naïve splenic B cells were cell surface biotinylated prior to pervanadate stimulation and SH2 domain adsorbent affinity purification. In this two-step enrichment protocol, binding partners were first enriched using SH2 domain adsorbents, with elution using low pH glycine buffer. Surface proteins were then isolated by adsorption to streptavidin agarose beads. Eluates were fractionated by SDS-PAGE and transferred to PVDF and analyzed by sequential blotting with anti-phosphotyrosine and streptavidin. Comparison of the SH2 domain affinity purified and 2-step enrichment by anti-pTyr blotting shows clearly that only a subset of binding partners are biotinylated cell surface proteins. Furthermore, reblotting with streptavidin reveals a similar repertoire although relative signal intensity of specific bands differs between pTyr and avidin blots. This may reflect the greater biotinylation of proteins that have larger extracellular domains, e.g. the 140Dda band relative to the ~60 kDa band (**Figure 5A**). Analysis of the relative capture of pCD79a, pFcγRIIB, and CD22 using the two protocols is consistent with this interpretation (**Figure 5B**). FcγRIIB which contains two linearly arranged extracellular Ig-like domains is sufficiently biotinylated to be captured by the avidin adsorbent. However, CD79a, containing only a single Ig-like domain, and its disulfide bonded partner CD79b also containing a single Ig-like domain, are not captured by avidin. This suggests that extension from the cell surface is a determinant of the utility of this approach. Specifically, 2-step enrichment may only be useful in identifying phosphatase binding partners that have large extracellular domains. The 2-step enrichment again confirms the selective purification of SH2 domain reagents and validity of this protocol to capture specific inhibitory receptors. Utilizing cell surface biotinylation and the 2-step enrichment described above allows for the purification of cell surface proteins for analysis *via* immunoblotting. The two-step approach is likely to be the most informative when combined with LC-MS/MS to define the SH2 domain-cell surface receptor interactome in an unsupervised manner.

## Discussion

The highly variable response of individuals to CBT in malignant disease underscores the importance of better understanding the inhibitory receptor landscape of immune system cells. However, identification of inhibitory receptors to be used as CBT therapeutic targets has historically been a challenge. Many known inhibitory immune receptors mediate their activity through the recruitment of inhibitory phosphatases *via* SH2 interactions with phosphorylated tyrosines in the receptor cytoplasmic tails. The approach taken here to identify novel inhibitory receptors was predicated on the assumption that additional as yet undefined inhibitory receptors exist, which utilizes this mechanism.

In this work we sought to develop a method, which we have termed IRT for Inhibitory Receptor Trap, to isolate and identify novel receptors that signal by engagement of the SH2 domains of inhibitory phosphatases (**Figure 1**). By targeting additional receptors, it should be possible to improve the efficacy of CBT. Our results indicate that SH2 domains of inhibitory phosphatases SHIP1, SHP1, and SHP2 can be used to effectively capture phosphorylated inhibitory receptors. We have shown that cell lines derived from different immune cell lineages (T cell, B cell, and myeloid) express unique repertoires of phosphatase SH2 domain binding receptors (**Figure 2D**). Utilizing *ex vivo* mouse splenic B cells and T cells, we showed that SH2 domain reagents from activating kinases bind common sets of proteins that are distinct from those of inhibitory phosphatases (**Figures 2A, B**). Differences are also seen in phosphoprotein sets bound by different phosphatases (**Figure 2D**). Furthermore, we observed binding of phosphatases to predicted partners including CD79a, FcγRIIB, CD22, and PD1 (**Figures 3A, B**). Our interest in defining receptor proteins led to the use of N-glycanase treatment in conjunction with immunoblotting as a tool to improve resolution of IRT (**Figure 3**). We demonstrated IRT effectiveness for capture of proteins, e.g., FcγRIIB and CD79a, phosphorylated as a consequence of specific receptor stimulation. Co-stimulation of these receptors using anti-IgG H+L induced sufficient phosphorylation to enable IRT capture of pFcγRIIB. However, stimulation by anti-H+L or F(ab’)_2_ anti-H+L led to capture of phosphorylated CD79a but not FcγRIIB (**Figure 4**). Finally, our experiments showed that cell surface biotinylation can be used in a 2-step purification approach to narrow IRT to reveal only surface receptors (**Figure 5**).

Interestingly, we noticed that in some cases multiple SH2 domain adsorbents bound apparently common phosphotyrosine-containing species in the same cell type and, in some cases, across cell types (**Figures 2A–D**). While we speculate based on size that these shared bands are the same proteins, without use of specific immunoblotting antibodies, we cannot make definitive conclusions. As shown in **Figure 2C**, when comparing B cells to T cells we noticed a band between the 25 and 40 kDa molecular weight markers (indicated by arrows) which is present in B but not T cell WCL and bound by multiple SH2 domains. The apparent identity of this band as CD79a was verified by anti-CD79a blot in **Figure 3A**.

The PD1 enrichment by SHIP1SH2 domains in pervanadate stimulated *ex vivo* splenic T cells was unexpected (**Figure 2E**). SHIP1 association with PD1 has not been reported by other groups who have explore PD1 binding partners (40). Interestingly a recent publication reported the dispensability of SHP2 in T cell exhaustion, it is possible that SHP2 and SHIP1 may play redundant roles in inhibitory PD1 signaling (37). In this context, it may be significant that the SHP2 (and SHP1) must be bound to their receptors to derepress phosphatase activity, while SHIP1 does not. In PD1 signaling, SHP2 would be expected to function locally dephosphorylating phosphotyrosines in reach, while SHIP1 may work broadly to reduce PtdIns3,4,5 levels globally inhibiting signaling in trans (23, 24, 27, 41).

Upon anti-H+L aggregation of antigen receptors on *ex vivo* splenic B cells, we observed predominant binding of phosphorylated FcγRIIB to SHIP1SH2 domain. There was significant, though lower, pFcγRIIB binding to the tandem SH2 domains of SHP1/2. This result is consistent with previous reports of SHP1 function in FcγRIIB signaling (**Figure 4C**) (10, 19, 42). We would speculate that SHP1/2 binding to FcγRIIB ITIM occurs when antigen receptors are highly crosslinked resulting in higher levels of FcγRIIB phosphorylation, as suggested by the pITIM binding experiments of Lesourne and Daeron (19).

Mono- versus bi-phosphorylation of conserved ITAM tyrosines serves as a molecular switch. Monophosphorylation leading to recruitment of Lyn which acts in both activating and inhibitory signaling, and bi-phosphorylation which enables Syk binding and activation (29, 38, 43). Y182 of CD79a is the predominantly phosphorylated of the two tyrosines in the ITAM motif (39) suggesting that Lyn, *via* its single SH2 domain, may bind CD79a at greater quantity than Syk during BCR signaling. It has been previously observed that Y193F mutation of CD79a has a minor effect on BCR induced phosphorylayion,while Y182F mutation nearly completely abrogates receptor induced phosphorylation (39). The greater pCD79a enrichment in IRT by LynSH2 compared to Syk(SH2)_2_ supports the notion that upon BCR stimulation CD79a is predominantly monophosphorylated, and this occurs on Y182.

Complementation of IRT with a filter that allows detection of only cell surface proteins yielded interesting and useful information. Avidin or phophosphotyrosine blotting of IRT enriched cell surface biotinylated pervanadate stimulated splenic B cells revealed a very bright band at ~150kD in both the SH2 domain and 2-step enrichment. This protein is likely CD22, and the strength of this signal probably relates to its large extracellular domain, mouse CD22 has a 681 amino acid extracellular domain, allowing greater biotinylation than small membrane proteins. For example, a lack of CD79a enrichment as seen in the 2-step enrichment when blotting for pTyr, avidin-HRP, and pCD79a (**Figures 5A, B**). This is presumably due to the smaller extracellular domain of CD79, of 109 amino acids, leading to reduced biotinylation. Furthermore, it is possible that associated immunoglobulin may mask some CD79 surfaces from biotinylation. Our data also shows 2-step enrichment clearly identifying pFcγRIIB which has a 181 amino acid extracellular domain and is not known to be constitutively associated with any other surface receptors (**Figure 5B**).

The fact that cell surface biotinylation may not enrich for receptors that have small extracellular domains or are closely associated with other receptors at the time of surface biotinylation should be a consideration in application of IRT.

In conclusion, our findings describe and justify the use of IRT for enrichment and identification of inhibitory receptors, which engage inhibitory phosphatases *via* phosphorylated ITIMs. We have developed IRT for use in an unsupervised manner with LC-MS/MS for the interrogation of cellular “inhibisomes” to better understand cellular inhibitory programming. We hope our method can be applied to CBT where there is an unmet need to identify new and novel inhibitory receptors to better tailor treatment of those suffering from malignant diseases.

## Data Availability Statement

The raw data supporting the conclusions of this article will be made available by the authors, without undue reservation.

## Ethics Statement

The animal study was reviewed and approved by University of Colorado Institutional Animal Care and Use Committee.

## Author Contributions

BC, RS, and VO performed the experiments. BC and VO analyzed the data. BC, IH, AG, and JC designed the experiments and wrote the paper. All authors contributed to the article and approved the submitted version.

## Funding

This work was supported by National Institutes of Health grants R01AI077597 (JCC), R01AI24487(JCC), R21AI149019 (AG) and by the Arthritis National Research Foundation (AG). IH was supported by NIAMS-5T32AR007534-32

## Conflict of Interest

The authors declare that the research was conducted in the absence of any commercial or financial relationships that could be construed as a potential conflict of interest.
