# Supplementary material for: Inhibitory Receptor Trap: A Platform for Discovery of Inhibitory Receptors That Utilize Inositol Lipid and Phosphotyrosine Phosphatase Effectors
Source: Front Immunol. 2020 Oct 21;11:592329. doi: 10.3389/fimmu.2020.592329 (PMC7641642; doi:10.3389/fimmu.2020.592329)
Supplement: Supplementary file 1 [file DataSheet_1.pdf]

Supplementary Figure 1

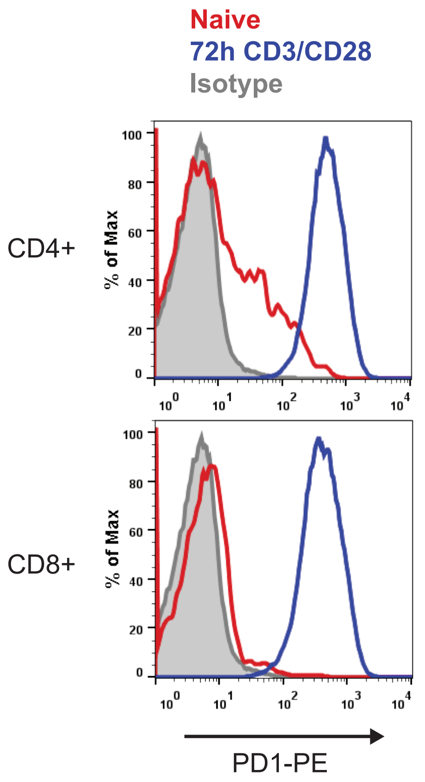

Supplementary Figure 1. Measurement of PD1 expression on ex vivo splenic T cells. After T cell activation was performed described in material and methods cells were stained with anti-CD4, anti-CD8, anti-PD1 or hamster Isotype. PD1 expression was measure via flow cytometry on BD LSR Fortessa X-20.
